# Sociodemographic correlates of discrimination against PLHIV in High HIV prevalence states of India, NFHS 2016–21

**DOI:** 10.1038/s41598-023-42162-6

**Published:** 2023-09-12

**Authors:** Shri Kant Singh, Neha Shri

**Affiliations:** Department of Survey Research and Data Analytics, International Institute for Population Sciences, Mumbai, Maharashtra 400088 India

**Keywords:** Health policy, Public health

## Abstract

This study investigates the socio-demographic correlates of HIV discrimination among individuals aged 15–49 years. This study also aims to assess the change in discriminatory attitudes towards PLHIV in high HIV prevalence states from 2016 to 2021 using data from the national Demographic Health Survey (4th and 5th). To identify factors associated with discriminatory attitudes, a multivariable logistic regression analysis was performed. Further, predicted probabilities and average marginal effects were computed, and the difference in discriminatory attitudes across both rounds was examined using a non-linear Fairlie decomposition. Mass media exposure, improved wealth index, and comprehensive knowledge significantly reduced the discriminatory attitudes towards PLHIV. Fairlie decomposition indicated that comprehensive knowledge, knowledge of mother-to-child transmission, and mass media exposure was significant contributor to the differences observed in the discriminatory attitude towards PLHIV across survey rounds. This study emphasizes the importance of spreading accurate information about HIV transmission modes and reinforces existing programmes and policies aimed at reducing stigma and discrimination against PLHIV. These programmes' efficiency and effectiveness can be ensured by linking them with community-level programmes and activities organized by Self Help Groups (SHGs), which have resulted in a paradigm shift in empowering women in India.

## Introduction

HIV/AIDS epidemic has not been just a medical condition but is also identified as a stigmatized illness^1^. Goffman^2^ defines stigma as an undesirable attribute of an individual that reduces his status in the eyes of society. Discrimination is a primary result of stigmatization and happens when someone is "treated unfairly and unjustly" because it is perceived that People living with HIV (PLHIV) are different from them. HIV stigma is documented to violate basic human rights and inhibits access to testing and care^3,4^. Although testing is crucial in reducing the risks of HIV infection, the increasing recognition of HIV/AIDS stigma as a factor that permeates multiple dimensions experienced with PLHIV is associated with a reduced likelihood of testing.

Abundant evidence is available on the discriminatory practices towards PLHIV across Asia in various domains such as health care, immigration, and migration laws. PLHIV faces discrimination in every area of social life such as from friends and families, in communities, and in educational and workplace settings. Previous research shows that the most covert and overt forms of discrimination PLHIV face are denied/delayed treatment in hospitals, disparaging remarks from family members or community, withdrawal of health/insurance benefits, physical isolation in hospitals and community, etc. ^5^. Researchers have reported discriminatory attitudes and behavior of service providers towards PLHIV which adversely impacts the quality of life of PLHIV^6–8^. Apart from people's ignorance, medical practitioners also stigmatize and discriminate against PLHIV^9^. Evidence highlights the stigmatization of HIV-infected individuals in Asia^10,11^ and India is no exception since an estimated 23.49 lakh people were living with HIV in the year 2019^12^.

Warwick and colleagues^13^ have categorized the factors associated with discrimination into individual and community-level factors. Factors such as age^14–16^, education^14–16^, gender^16^, marital status^17^, wealth status^18^, mass media exposure^19^ determine their social acceptance an individual level. Moreover, studies have assessed the community-level factors that affect the acceptance of PLHIV in a community^20^. Evidence highlights that ideas, resources, and behavior of people residing in the community predict individual behavior and knowledge^21^. People gossip, insult, and physically assault them in communities and healthcare settings^22^. The belief that PLHIV is a shame for communities and families results in their abandonment, banishing, hiding, and even murder^23,24^. Further, social capital has also been recognized as a factor affecting HIV–related outcomes including HIV stigma and unprotected sexual intercourse^25^. Social capital is found to be associated with a lower risk of HIV by facilitating condom use^26^, HIV testing, and reduced HIV stigma and discrimination^27^.

Studies conducted across the country have reported a high prevalence of discrimination against PLHIV among healthcare providers^28,29^and its association with treatment delays and poor health outcomes in southern Indian states^30,31^. At the national level, around 0.21% of the adults were infected with HIV/AIDS in 2021 with northern and southern states bearing the highest adult HIV prevalence. An overview of India’s progress towards the testing and treating targets of 95-95-95 indicates that 77% of PLHIV know their HIV status out of which 65% are receiving ART and only 55% of them have viral load suppression^32^. Despite substantial improvements in antiretroviral therapy (ART) and numerous efforts towards increasing knowledge and awareness regarding prevention, negative attitudes towards PLHIV are a barrier to achieving the goal of ending AIDS as a public health threat by 2030^33^.

Accepting attitudes toward PLHIV has been documented to be associated with individual-level characteristics^34^. Further researchers have highlighted the importance of community wealth in improving the accepting attitudes towards PLHIV^34,35^. Despite the high prevalence of HIV and the negative consequences of stigma and discrimination towards PLHIV, there is a paucity of research partly due to the heterogeneous population in terms of religion and socio-cultural settings. Considering the paramount implications of discrimination on health policies and programs aimed at effective prevention and treatment, this study examines the socio-demographic correlates of discrimination towards PLHIV in the country. Additionally, we have aimed to assess the magnitude of changes in discriminatory attitudes across the half decade and differences in HIV discrimination across socio-economic groups.

## Materials and methods

### Data

This study utilizes data from the fourth and fifth series of the National Family Health Survey (NFHS) conducted during 2015–16 and 2019–21 respectively. The NFHS surveys are part of global Demographic and Health Surveys, conducted under the stewardship of the Ministry of Health and Family Welfare (MoHFW), Government of India with the International Institute for Population Sciences (IIPS), Mumbai, designated as the nodal agency. This survey provides information on health and family welfare, and sexual behavior with vital estimates through a series of biomarker tests and measurements at the state and national levels. NFHS sample is a stratified two-stage sample that used the 2011 census as the sampling frame for the selection of PSUs. Detailed information on the data, survey designs, sampling frame, and quality check measures have been presented in the national report of NFHS-4^36^. The data set is available on the DHS website and therefore does not require any ethical approval.

### Selection of the study sample

The survey generated different datasets. We used the individual record file for men and women separately that captured diverse information on knowledge and attitudes towards HIV/AIDS. The effective sample size for the present study was 21,454 women and 19,047 men from the 4th round and 21,675 women and 19,561 men aged 15–49 years from the 5th round of the survey. Those respondents who never heard of HIV/AIDS were dropped from the sample. The analysis has been restricted to eight high HIV prevalence states namely Nagaland, Manipur, Mizoram, Tamilnadu, Maharashtra, Andhra Pradesh, Karnataka, and Telangana. These states bear a significantly higher burden of HIV with the HIV prevalence higher than the national average^37^.

### Variable description

#### Outcome variable

The outcome variable for the study was the discriminatory attitudes of respondents towards people living with HIV. The respondents who have heard of HIV were asked the following questions.

- Would you buy fresh vegetables from a shopkeeper or vendor if you knew that this person had HIV?
- Do you think that children living with HIV should be able to attend school with children who are HIV-negative?

Those who responded “no” to either of these two questions were recoded to have discriminatory attitudes.

#### Exposure variables

The main exposure variables were mass media exposure and comprehensive knowledge. Respondents were asked how often they read the news, listen to the radio or watch television. Those who responded at least once a week were considered to have regular exposure to mass media. The respondent was categorized as having a comprehensive knowledge of HIV if he knew that the consistent use of condoms during sexual intercourse and having just one uninfected faithful partner can reduce the chances of getting HIV/AIDS and knowing that a healthy-looking person can have HIV/AIDS, and rejecting two common misconceptions about transmission or prevention of HIV/AIDS. The two common misconceptions of HIV /AIDS are ‘HIV can be transmitted by mosquito bites’ and ‘a person can become infected by sharing food with a person who has HIV/AIDS’.

Age was categorized as 15–29 years, 29–39 years and 40–49 years. Educational status was categorized as no formal education/ primary not completed, primary, secondary, and higher. Respondent’s marital status was categorized as never in a union, currently in a union, and formerly in a union. The wealth quintile was assessed based on the number and kinds of consumer goods they own, ranging from a television to a bicycle or car, and housing characteristics such as the source of drinking water, toilet facilities, and flooring material. Further, these scores were derived using principal component analysis and the households were divided into different wealth quintiles i.e. poorest, poorer, middle, richer and richest. Religion was coded as Hindu, Muslim, Christian, and Others. Caste was recoded as Scheduled Tribe (ST), Scheduled Caste (SC), Other Backward Class (OBC), and others. The place of residence was categorized as rural and urban. To assess knowledge of mother-to-child transmission, respondents were asked whether HIV can be transmitted from a mother to her child during pregnancy, during delivery, and by breastfeeding, and their response was recorded. The respondents were asked if they used a condom during their last sex with their most recent partner and their responses were recoded as yes or no.

### Statistical analysis

Descriptive statistics and bivariate analysis have been performed to determine the prevalence of discriminatory attitudes by socio-economic status. Logistic regression analysis was used to understand the factors determining the prevalence of discriminatory attitudes. Predicted probabilities were computed to understand the magnitude of changes in the discriminatory attitudes among men and women in the half decades i.e. from NFHS-4 to NFHS-5 after adjusting the effects of some socio-demographic and contextual characteristics. To analyze the gap in the determinants of discrimination and the extent to which they explain the differences in HIV discrimination across socio-economic groups, Fairlie decomposition has been used^38^. The cause of the gap between discriminatory attitudes in the two rounds of the survey is examined using a non-linear decomposition. The positive contribution of a covariate indicated that the variable contributed to the widening of the discriminatory attitudes in the two rounds of the survey and the negative coefficient indicated that the variable helped in reducing the gap in the two rounds of the survey. The coefficients have been presented in terms of percentage. To adjust for the non-proportional allocation of the sample to the different survey domains, sampling weights have been applied ensuring the actual representativeness of the survey results at the national level and as well as at the domain level. The whole statistical analysis was performed by using STATA version 16^39^.

### Consent to participate

This study uses data from secondary sources and thus no consent was required. However, informed consent was taken from eligible participants and the purpose of the survey was explained to them.

## Results

The background characteristics of the eligible respondents are presented in Table 1. Very few percent of the population did not have access to mass media and mass media exposure was higher among males. However, the proportion of individuals with mass media exposure was lower in NFHS-5 than in the previous survey. A higher percentage of the eligible respondents were from middle and richer wealth quintiles and resided in rural areas. Despite an increase in the percentage of women having comprehensive knowledge regarding HIV/AIDS from NFHS-4 to NFHS-5, comprehensive knowledge was very low among women (25% in NFHS-4 and 31% in NFHS-5). However, the proportion of men having comprehensive knowledge regarding HIV/AIDS has marginally improved. Further, the level of comprehensive knowledge was higher among men than among women. Additionally, only 57% of men and 67% and 75% (NFHS-4 & 5 resp.) of the women had correct knowledge of ways of HIV/AIDS transmission from the mother to child. A majority of the respondents reported not using a condom at the last sex in both rounds of the survey.

**Table 1:** Descriptive characteristics of samples by socio-economic characteristics in high HIV prevalence states of India, 2016–21.

Covariates	Men				Women			
	NFHS-4		NFHS-5		NFHS-4		NFHS-5	
	Frequency	%	Frequency	%	Frequency	%	Frequency	%
*Age (in years)*								
15–24	5989	31.18	5698	30.40	6925	32.62	6122	28.07
25–39	8566	45.17	9014	45.55	9666	44.32	9979	46.61
40 and above	4492	23.66	4849	24.05	4863	23.07	5574	25.31
*Educational status*								
No formal Education	1551	8.93	1610	8.39	3194	17.25	3635	15.78
Primary	1984	9.7	1793	8.95	2506	10.93	2267	9.85
Secondary	11,769	58.65	11,803	57.82	12,465	53.85	11,972	53.88
Higher	3743	22.72	4355	24.84	3289	17.97	3801	20.49
*Marital status*								
Never in union	7570	38.47	7797	40.17	5289	21.44	4900	21.07
Currently in union	11,235	60.57	11,526	58.81	14,918	72.75	15,345	72.52
Formerly in union	242	0.96	238	1.02	1247	5.8	1430	6.42
*Mass media exposure*								
No exposure	1343	4.72	4954	22.27	2274	7.31	6130	23.3
Mass media Exposure	17,704	95.28	14,607	77.73	19,180	92.69	15,545	76.7
*Wealth quintile*								
Poorest	1037	3.97	1714	5.61	1057	3.42	1808	4.94
Poorer	3735	14.55	3880	15.3	3819	13.37	4254	14.98
Middle	5476	25.84	5597	25.66	6091	25.92	6271	26.87
Richer	5339	30.45	5401	29.73	6260	30.9	5878	29.54
Richest	3460	25.19	2969	23.70	4227	26.38	3464	23.67
*Religion*								
Hindu	12,869	83.07	14,290	82.64	67.06	82.84	15,685	84.05
Muslim	1660	10.25	1602	10.14	8.51	9.88	1736	8.71
Christian	3802	3.72	3007	3.39	20.97	4.44	3609	4.38
Others	716	2.96	662	3.83	3.46	2.84	645	2.86
*Caste*								
Scheduled Caste (SC)	3246	19.8	3484	19.60	3789	20.13	4013	21
Scheduled Tribes (ST)	4422	6.65	4070	8.44	4909	6.56	4545	7.65
Other Backward Castes (OBC)	7788	48.86	8785	46.96	9033	50.1	10,003	51.72
Others	3591	24.7	3222	25.00	3723	23.2	3114	19.63
*Residence*								
Urban	7647	48.7	6433	43.83	8972	48.54	7259	43.44
Rural	11,400	51.3	13,128	56.17	12,482	51.46	14,416	56.56
*Comprehensive knowledge*								
No	11,965	63.24	12,471	62.46	15,706	75.12	14,932	69.4
Yes	7082	36.76	7090	37.54	5748	24.88	6743	30.6
*Knowledge of mother-to-child transmission*								
No	8502	43.19	7804	42.56	7759	32.5	5484	24.78
Yes	10,545	56.81	11,757	57.44	13,695	67.5	16,191	75.22
*Used condom at last sex*								
No	18,109	93.99	18,254	91.25	20,717	95.42	20,733	94.42
yes	938	6.01	1307	8.75	737	4.58	942	5.58
*State*								
Andhra Pradesh	1349	13.78	1350	16.24	1668	14.73	1498	14.48
Karnataka	3296	15.41	4016	22.84	3410	14.75	4404	19.6
Maharashtra	4,152	32.67	4723	42.04	3967	27.79	4430	31.9
Manipur	1740	0.51	1062	0.75	2090	0.56	1248	0.74
Mizoram	1595	0.25	999	0.33	1884	0.28	1128	0.33
Nagaland	1343	0.31	1254	0.43	1490	0.33	1459	0.43
Tamilnadu	4560	26.98	2935	6.90	5797	31.43	3708	22.9
Telangana	1012	10.09	3222	10.47	1148	10.13	3800	9.62
Total	19,047		19,561		21,454		21,675	

Results from Table 2 indicate the prevalence of discriminatory attitudes to different socio-demographic characteristics. Around 29% of men in NFHS-4 had discriminatory attitudes towards PLHIV and it increased marginally in NFHS-5 (30%). Similarly, 37% of women had discriminatory attitudes towards PLHIV which increased by 5% points in NFHS-5. The discriminatory attitude was found to be higher among older individuals, those without any formal education and poor wealth, Over the period 2016–21, the discriminatory attitude was low among individuals having mass media exposure in comparison to men and women without any mass media exposure. As we move from the poorest to the richest wealth quintile, the percentage of individuals having a discriminatory attitude towards PLHIV is reduced. A higher percentage of rural men (33%) and women (44%) had discriminatory attitudes than urban men(25%) and women (39%) in NFHS-5. Moreover, a lower percentage of men and women having comprehensive knowledge displayed discriminatory attitudes than their counterparts without comprehensive knowledge of HIV/AIDS (41% vs. 46% in NFHS-5 among women). Prevalence of discriminatory attitudes was found to be highest among women and men from Tamilnadu followed by Telangana in NFHS-5 and least in Manipur & Mizoram in NFHS-4 & 5.

**Table 2:** Prevalence of discriminatory attitudes by socio-economic characteristics in high HIV prevalence states of India, 2016–21.

Characteristics	Men		Women	
	NFHS-4	NFHS-5	NFHS-4	NFHS-5
*Age (in years)*				
15–24	28.28	31.62	34.07	42.55
25–29	28.08	26.57	36	39.16
30–34	32.32	32.93	41.65	46.76
*Educational status*				
No formal education	43.91	42.02	51.24	51.22
Primary	36.7	39.33	44.06	48.76
Secondary	29.44	30.97	35.4	41.73
Higher	19.35	18.85	22.38	32.55
*Marital status*				
Never in union	27.33	29.3	32.25	39.48
Currently in union	30.34	29.85	37.85	42.61
Formerly in union	26.28	30.7	39.43	43.93
*Mass media exposure*				
No exposure	40.81	33.44	47.59	43.87
Mass media exposure	28.56	28.55	35.88	41.48
*Wealth quintile*				
Poorest	41.05	40.83	46.16	51.43
Poorer	34.72	37.09	45.2	46.64
Middle	31.28	32.44	41.25	43.76
Richer	28.12	28.12	35.84	42.17
Richest	23.09	21.04	27.85	35.05
*Religion*				
Hindu	29.17	30.12	37.49	42.91
Muslim	29.85	30.52	34.5	40.24
Christian	28.37	24.18	34.25	38.46
Others	26.87	21.78	26.4	27.34
*Caste*				
Scheduled Caste (SC)	30.93	32.08	40.14	43.55
Scheduled Tribes (ST)	33.43	35.6	40.68	44.15
Other Backward Caste (OBC)	30.92	30.22	37.74	45.49
Others	23.05	24.61	30.5	30.51
*Residence*				
Urban	26.99	25.08	33.19	38.92
Rural	31.19	33.19	40.08	44.43
**Co***mprehensive knowledge*				
No	36.41	36.21	42.67	48.6
Yes	16.64	18.69	18.82	27.15
*Knowledge of mother-to-child transmission*				
No	30.82	31.34	39.93	46.26
Yes	27.87	28.37	35.2	40.64
*Used condom at last sex*				
No	29.54	30.28	37.07	42.66
Yes	22.99	22.92	29.76	31.45
*State*				
Andhra Pradesh	25.39	29.21	36.23	38.63
Karnataka	24.74	26.96	38.71	36.35
Maharashtra	23.2	27.51	29.11	30.79
Manipur	17.04	16.33	21.33	19.51
Mizoram	12.41	10.29	13.25	13.68
Nagaland	46.76	23.3	50.46	29.58
Tamilnadu	36.92	52.14	41.69	64.97
Telangana	39.92	31.68	41.24	44.71
Total	29.14	29.64	36.74	42.04

Results presented in Table 3 portray the results obtained from logistic regression analysis of discriminatory attitudes by socio-economic status. The odds of having discriminatory attitudes were reduced by half as moved from men and women without any formal education to those with higher levels of education for both rounds. For instance, in NFHS-4, men with higher levels of education were 52% less likely to have discriminatory attitudes than men without any formal education [OR 0.48, CI 0.41–0.55]. In NFHS-5, women with higher levels of education were 56% less likely to have discriminatory attitudes than women without any formal education [OR:0.44, CI 0.39–0.50]. Exposure to mass media significantly reduced the risk of having discriminatory attitudes among men and women in NFHS-4. However, mass media was not significantly associated with discriminatory attitudes in the latest survey round. Rural place of residence was associated with an increased likelihood of having discriminatory attitudes among women in NFHS-4 and men in NFHS-5. Additionally, having comprehensive knowledge reduced the risk of having discriminatory attitudes by 48% and 56% for men and women respectively [OR 0.52, CI 0.48–0.56; OR 0.44, CI 0.41–0.47 in NFHS-5]. Similarly, men and women with knowledge of mother-to-child transmission had lower odds of having discriminatory attitudes than individuals without correct knowledge of mother-to-child transmission. Men and women from Tamilnadu were 2.95 times and 3.69 times respectively more likely to have discriminatory attitudes than their respective counterparts from Andhra Pradesh [OR 2.95, CI 2.55–3.41 and OR 3.69, CI 3.23–4.21; NFHS-5].

**Table 3:** Results from logistic regression analysis of discriminatory attitudes by socio-economic characteristics in high HIV prevalence states in India, 2016–21.

Characteristics	NFHS-4		NFHS-5	
	Men	Women	Men	Women
*Age (in years)*				
15–24®				
25–39	0.87*** (0.78–0.96)	0.88*** (0.81–0.96)	0.76*** (0.69–.84)	0.75*** (0.69–0.82)
40 and above	1 (0.88–1.14)	0.97 (0.88–1.08)	0.82*** (0.72–0.93)	0.81*** (0.73–0.9)
*Educational status*				
No formal education®				
Primary	0.87* (0.75–1)	0.91* (0.81–1.01)	0.91 (0.79–1.05)	0.89** (0.79–0.99)
Secondary	0.67*** (0.6–0.76)	0.68*** (0.62–0.74)	0.72*** (0.64–0.81)	0.69*** (0.63–0.76)
Higher	0.48*** (0.41–0.55)	0.45*** (0.39–0.51)	0.48*** (0.41–0.55)	0.44*** (0.39–0.5)
*Marital status*				
Never in union®				
Currently in union	1.07 (0.96–1.19)	1.09* (1–1.2)	1.09 (0.98–1.2)	1.15*** (1.04–1.26)
Formerly in union	0.95 (0.69–1.33)	1.01 (0.86–1.18)	1.06 (0.78–1.44)	1.16* (0.99–1.34)
*Mass media exposure*				
No exposure®				
Mass media exposure	0.68*** (0.6–0.77)	0.79*** (0.71–0.88)	0.98 (0.91–1.06)	1.01 (0.95–1.09)
*Wealth quintile*				
Poorest®				
Poorer	0.78*** (0.67–0.91)	0.91 (0.79–1.05)	0.78*** (0.69–0.89)	0.87** (0.77–0.98)
Middle	0.7*** (0.6–0.82)	0.84** (0.73–0.97)	0.69*** (0.61–0.79)	0.8*** (0.71–0.91)
Richer	0.6*** (0.52–0.71)	0.74*** (0.63–0.86)	0.65*** (0.57–0.74)	0.82*** (0.72–0.93)
Richest	0.57*** (0.47–0.67)	0.69*** (0.59–0.82)	0.59*** (0.51–.69)	0.75*** (0.64–0.87)
*Religion*				
Hindu®				
Muslim	1.09 (0.96–1.23)	0.97 (0.87–1.08)	1.07 (0.94–1.2)	1.1* (0.99–1.23)
Christian	1.02 (0.86–1.2)	1.02 (0.89–1.18)	0.95 (0.79–.13)	1.01 (0.88–1.17)
Others	1.21* (0.99–1.48)	1.06 (0.88–1.27)	0.92 (0.75–1.13)	1.12 (0.92–1.37)
*Caste*				
Scheduled Caste (SC)®				
Scheduled Tribes (ST)	1.24*** (1.07–1.44)	1.16** (1.02–1.33)	1.07 (0.93–1.22)	1.31*** (1.16–1.48)
Other Backward Caste (OBC)	1.1* (1–1.21)	1.01 (0.93–1.09)	0.97 (0.89–1.06)	1.11** (1.03–1.21)
Others	1.1 (0.97–1.25)	1 (0.89–1.12)	0.81*** (0.72–0.91)	1 (0.89–1.12)
*Residence*				
Urban®				
Rural	1.06 (0.98–1.15)	1.07** (1–1.15)	1.07* (0.99–1.16)	1.03 (0.96–1.11)
*Comprehensive knowledge*				
No®				
Yes	0.4*** (0.37–0.43)	0.39*** (0.36–0.43)	0.52*** (0.48–0.56)	0.44*** (0.41–0.47)
*Knowledge of mother-to-child transmission*				
No®				
Yes	0.87*** (0.81–0.93)	0.88*** (0.83–0.94)	0.87*** (0.82–0.93)	0.83*** (0.78–0.89)
*Used condom at last sex*				
No®				
Yes	0.92 (0.78–1.08)	1.01 (0.86–1.19)	0.91 (0.79–1.04)	0.95 (0.82–1.1)
*State*				
Andhra Pradesh	0.72*** (0.61–0.84)	1.2*** (1.06–1.37)	0.91 (0.79–1.05)	0.98 (0.86–1.11)
Karnataka	0.89 (0.77–1.04)	0.87** (0.76–0.99)	1.2** (1.04–1.39)	0.87** (0.76–0.99)
Maharashtra	0.53*** (0.44–0.65)	0.51*** (0.43–0.61)	0.58*** (0.46–0.73)	0.44*** (0.36–0.54)
Manipur	0.45*** (0.35–0.57)	0.42*** (0.34–0.52)	0.37*** (0.28–0.5)	0.29*** (0.22–0.37)
Mizoram	1.51*** (1.2–1.9)	1.49*** (1.22–1.82)	0.66*** (0.51–0.85)	0.56*** (0.45–0.69)
Nagaland	1.2** (1.03–1.39)	1.3*** (1.15–1.47)	2.95*** (2.55–3.41)	3.69*** (3.23–4.21)
Tamilnadu	2.02*** (1.68–2.42)	1.49*** (1.26–1.74)	1.25*** (1.08–1.4)	1.25*** (1.1–1.42)
Telangana	1.65 (1.28–2.13)	1.49 (1.2–1.85)	0.8 (0.59–1.07)	1.41*** (1.15–1.72)

Note: ® represents reference category. *Represents statistically significant values at 90% CI. **represents statistically significant values at 95% CI. ***represents statistically significant values at 99% CI.

Table 4 depicts the predicted probability of discriminatory attitudes of men and women towards PLHIV estimated concerning socio-demographic characteristics. The predicted probability of having discriminatory attitudes by wealth quintile witnessed an increase in the period 2016–21 for both sexes. However, as we moved from the poorest to the richest wealth quintile, there was a reduction in the predicted probability of having discriminatory attitudes. The predicted probability of discriminatory attitudes declined by 3.7 percent and 0.4 percent among men and women without any exposure to mass media during 2016–21. However, the predicted probability of having discriminatory attitudes among males with comprehensive knowledge increased from 0.16 to 0.53 in 2016–21. For the females, this predicted probability has increased from 0.05 in NFHS-4 to 0.06 in NFHS-5. The predicted probability of having a discriminatory attitude was reduced in the period 2016–21 for men and women from urban area and rural areas without mass media exposure. The predicted probability of having discriminatory attitudes was 33.6% and 34.4% in 2016 for men from urban and rural areas without comprehensive knowledge which increased marginally to 34.5% and 36% in 2021. However, for females without comprehensive knowledge irrespective of place of residence the predicted probability was around five percent in the period 2016–21.

**Table 4:** Predicted probability of discriminatory attitudes with respect to wealth index, mass media exposure, residence and comprehensive knowledge in high HIV prevalence states of India, 2016–21.

	Male		Change in Predicted Prob	Female		Change in Predicted Prob
	NFHS-4 (2015–16)	NFHS-5 (2020–21)	(2016–2022)	NFHS-4 (2015–16)	NFHS-5 (2020–21)	(2016–2021)
	Predicted Prob [CI]	Predicted Prob [CI]		Predicted Prob [CI]	Predicted Prob [CI]	
*Wealth index*						
Poorest	0.36 (0.33–0.39)	0.385 (0.36–0.41)	0.025	0.407 (0.38–0.44)	0.443 (0.42–0.47)	0.036
Poorer	0.31 (0.3– 0.33)	0.334 (0.32–0.35)	0.024	0.386 (0.37–0.4)	0.413 (0.4–0.43)	0.027
Middle	0.29 (0.28–0.3)	0.31 (0.3–0.32)	0.020	0.37 (0.36–0.38)	0.397 (0.39–0.41)	0.027
Richer	0.262 (0.25–0.27)	0.297 (0.29–0.31)	0.035	0.342 (0.33–0.35)	0.4 (0.39–0.41)	0.058
Richest	0.251 (0.23–0.27)	0.28 (0.26–0.3)	0.029	0.329 (0.31–0.35)	0.381 (0.36–0.4)	0.052
*Mass media exposure*						
No	0.353 (0.33–0.38)	0.316 (0.3–0.33)	− 0.037	0.404 (0.38–0.42)	0.4 (0.39–0.41)	-0.004
Yes	0.278 (0.27–0.28)	0.313 (0.31–0.32)	0.035	0.354 (0.35–0.36)	0.403 (0.4–0.41)	0.049
*Comprehensive knowledge*						
No	0.34 (0.33–0.35)	0.356 (0.35–0.36)	0.016	0.403 (0.4–0.41)	0.453 (0.44–0.46)	0.050
Yes	0.177 (0.17–0.19)	0.23 (0.22–0.24)	0.053	0.218 (0.21–0.23)	0.282 (0.27–0.29)	0.064
*Residence & mass media exposure*						
Urban w/o MME	0.38 (0.33–0.43)	0.321(0.3–0.35)	− 0.059	0.388 (0.35–0.43)	0.41 (0.39–0.43)	0.022
Urban with MME	0.269 (0.26–0.28)	0.298 (0.28–0.31)	0.029	0.344 (0.33–0.36)	0.397 (0.38–0.41)	0.053
Rural w/o MME	0.365 (0.33–0.39)	0.32 (0.3–0.34)	− 0.045	0.422 (0.4–.45)	0.402 (0.39–0.42)	− 0.020
Rural with MME	0.282 (0.27–0.29)	0.318 (0.31–0.33)	0.036	0.359 (0.35–0.37)	0.405 (0.4–0.41)	0.046
*Residence & comprehensive knowledge*						
Urban w/o knowledge	0.336 (0.32–0.35)	0.345 (0.33–0.36)	0.009	0.393 (0.38–0.41)	0.442 (0.43–0.46)	0.049
Urban with knowledge	0.164 (0.15–0.18)	0.224 (0.21–0.24)	0.060	0.213 (0.19–0.23)	0.291 (0.27–0.31)	0.078
Rural w/o knowledge	0.344 (0.33–0.36)	0.361 (0.35–0.37)	0.017	0.411 (0.4–0.42)	0.457 (0.45–0.47)	0.046
Rural with knowledge	0.184 (0.17–0.2)	0.233 (0.22–0.25)	0.049	0.22 (0.2– 0.24)	0.276 (0.26–0.29)	0.056

The results of the Fairlie decomposition of the difference in discriminatory attitudes between various socio-economic groups are presented in Table 5. Results indicated that approximately, 75% of the disparity in discriminatory attitudes across two survey rounds among men and 15% of disparity among women was explained by differences in these predictors. Mass media exposure is the most significant contributor to the widening difference in the discriminatory attitude towards PLHIV between NFHS-4 and NFHS-5 for males (86%) and females (156%) followed by wealth index. On the other hand, comprehensive knowledge and knowledge of mother-to-child transmission narrowed the gap between discriminatory attitudes in two rounds of the survey across both sexes. Other indicators such as place of residence (6% among men and 4% among women), played a significant role in the widening gap in the discriminatory attitudes towards PLHIV in the period 2016–21. Indicators like marital status, religion, and using a condom at last sex significantly contributed to narrowing the gap in discriminatory attitudes towards PLHIV in two rounds of the survey.

**Table 5:** Results of non-linear Fairlie decomposition of discriminatory attitudes in high HIV prevalence states of India, 2015–16 to 2019–21.

Characteristics	Men	Women
	% contribution	% contribution
Age	0.51	− 4.20
Education	− 7.98	8.55
Marital status	0.03	1.52
Mass media exposure	86.04	155.95
Wealth index	14.44	44.62
Religion	2.21	29.41
Caste	− 0.28	4.04
Place of residence	5.49	25.62
Comprehensive knowledge	2.54	− 149.31
Knowledge of mother-to child transmission	− 3.59	− 27.54
Used condom at last sex	− 1.16	− 0.02
State	1.85	11.48
Difference	− 0.0296	0.0433
Total explained	− 0.0222	− 0.0064
Number of observations	38,608	43,129

## Discussion

Using the nationally representative sample, the study aimed to identify the factors affecting the discrimination towards PLHIV in high HIV prevalence states of India and to examine the changes over time. Despite the government ban on discriminatory attitudes towards PLHIV, the change in discriminatory attitudes has changed marginally in the half-decade for men and women. The shortage of HIV/AIDS awareness programs in recent years might have led to the non-so-significant change in discriminatory attitudes. Moreover, India’s concentrated efforts in sub-groups of the population identified as high-risk groups have left the engagement of every individual thus accounting for the low and insignificant changes. This might have a substantial impact on emotional well-being, healthcare-seeking behavior, and disclosure of HIV status among those infected with HIV^40,41^ and thus fueling the spread of HIV.

The result clearly showed that the discriminatory attitude towards PLHIV is significantly associated with individual-level characteristics such as age, educational status, marital status, mass media exposure, wealth quintile, religion, caste, residence, and comprehensive knowledge. This is consistent with previous studies^34,35^. We observed that in NFHS-4, younger men and women were less likely to have discriminatory attitudes however, in the recent round of the survey, younger men and women were more likely to have discriminatory attitudes. Previous studies which have reported higher stigma among younger individuals^42,43, 44^ are of the view that young individuals have erroneous beliefs about the modes of transmission and prevention practices leading to stigma and discrimination towards PLHIV. Past studies have highlighted the importance of HIV-related information on prevention and control services in reducing discriminatory attitudes in India^42^. Similar to our findings, a study conducted in Tajikistan found that women of reproductive age experienced a considerable decline in their capacity to correctly identify transmission beliefs and prevention strategies in the period 2000–05^45^.

Our finding that education is a significant predictor in reducing discriminatory attitudes i.e. the risk of having discriminatory attitudes decreased with higher educational attainment was in line with previous studies^46–48^. Illiteracy might result in having a misconception about correct modes of transmission and consequently resulting in having discriminatory attitudes towards PLHIV. Results indicate that despite having an increase in the comprehensive knowledge about HIV/AIDS over the two survey rounds, the discriminatory attitude among men has remained constant. Although numerous studies have reported that exposure to mass media reduces the risk of having a discriminatory attitude towards PLHIV and results obtained from 2015 to 16 are in line with them^46,49^, results from the recent round of the survey were in contrast. One of the probable reasons behind higher discriminatory attitudes among younger individuals and higher discriminatory attitudes despite increased mass media in the recent round might be the reduced government’s focus on awareness regarding HIV/AIDS. The massive counseling and test and treatment policy in the past might have helped individuals gain HIV-related information and reduced negative attitudes about HIV-infected people.

Our result that individuals with higher economic status had a lower likelihood of having discrimination is consistent with studies conducted in China and Kenya^34,35^ and predicted the probability of having discriminatory attitudes reduced from poorest to richest wealth quintiles. Consistent with previous literature, we found that rural place of residence was associated with an increased likelihood of having discriminatory attitudes^46,48^. Access to mass media and various health promotion messages particularly in urban areas might contribute to this rural–urban divide in discriminatory attitudes. Additionally, having comprehensive knowledge reduced the risk of having discriminatory attitudes by 49% and 16% for men and women respectively [OR 0.51, CI 0.46–0.56 in NFHS-4; OR 0.84, CI 0.76–0.91 in NFHS-5]. Results from Fairlie's decomposition indicated that mass media exposure and comprehensive knowledge significantly had the largest contribution to discriminatory attitudes for both sexes. In terms of comprehensive knowledge, correct information may be crucial for both promoting acceptance of PLHIV and reducing discriminatory attitudes towards PLHIV. Studies have highlighted the role of mass media campaigns in increasing HIV knowledge and decreasing the gaps in related stigmatization and discriminatory behavior.

Surprisingly, knowledge regarding mother-to-child transmission was associated with an increased risk of having discriminatory attitudes and the risk was very high for women. Using a condom at the last sex was significantly associated with an increased risk of discriminatory practices for women in this study. This is in contrast with a study reporting a negative association between condom use and perceived stigmatization^42^. Results obtained from Fairlie decomposition also indicated that correct knowledge of mother-to-child transmission and using a condom at the last sex contributed mostly to this difference. Men and women from Meghalaya were 2.68 times and 2.56 times respectively more likely to have discriminatory attitudes than their respective counterparts from Nagaland. Although Meghalaya has a lower HIV prevalence than Nagaland (India HIV Estimation 2017), the lower level of comprehensive knowledge and ways of HIV transmission might have contributed to higher discriminatory attitudes in Nagaland. It is well established that the type of epidemic experienced in a particular setting can influence an individual’s attitudes^50^. As far as the coverage is concerned, it is important to make efforts to improve the coverage in high-priority states.

Overall, the predicted probabilities have revealed that the probability of having a discriminatory attitude towards PLHIV has increased marginally. Accepting attitudes are often affected by individual-level and community-level experiences^34^. The largest increment in the probability of having a discriminatory attitude was observed among men with comprehensive knowledge, from the poorest wealth index, and those with mass media exposure. This may be attributed to the striking improvement in media exposure irrespective of educational attainment.

Previous cohort and cross-sectional studies have found that actual or perceived prejudice can lead to emotions of worthlessness and self-blame from a variety of sources, including healthcare professionals, community members, and intimate relationships, which can eventually undermine adherence to ART regimens^51,52^. Moreover, intersectional stigma has been directly linked to decreased ART adherence, and racial discrimination is associated with having a detectable viral load^53^. Thus, discrimination not only affects an individual’s mental health but also hinders their access to healthcare services, resulting in PLHIV remaining underground.

## Conclusions and recommendations

Despite recent AIDS education campaigns that have increased public awareness of HIV/AIDS, the marginal increase in the discriminatory attitude towards PLHIV in high HIV prevalence states of India is a matter of concern. Therefore, the empirical evidence of the magnitude of discriminatory attitudes and practices towards PLHIV in the country along with its key drivers in high HIV prevalence states may work as vital inputs in designing suitable interventions to enhance accepting attitudes to PLHIV. This may help enhance service uptakes by PLHIV and change their health-related quality of life. The findings advocate for strengthening and expanding HIV testing and treatment services conjointly imparting accurate information about HIV transmission modes. Findings portray the need to reinforce existing programmes and policies and promote information campaign policies aimed at reducing discrimination against PLHIV. It is also critical to increase access to HIV counseling and testing services, as well as to develop educational initiatives addressing misconceptions about modes of transmission to combat discrimination. These programmes' efficiency and effectiveness can be ensured by linking them with community-level programmes and activities organized by Self Help Groups (SHGs), which has resulted in a paradigm shift in empowering women in India. Since discrimination against PLHIV can have a negative impact on their mental health and access to healthcare services, it is important to address discrimination as part of the overall HIV prevention and control strategy.

### Limitations

One of the major limitations that this study faces is the self-reported information on discriminatory attitudes which might include errors and biases. Moreover, the questions used for assessing the discriminatory attitudes towards HIV/AIDS were hypothetical. Thus, what people reported and what they might do is not clear. Thus, the direct relationship between attitudes and behaviors could not be established. The cross-sectional nature of this prevents us from establishing cause-and-effect relationships and result interpretation is just an association.

## Data Availability

The data used in this study is available freely in the public domain at the Demographic and Health Surveys (DHS) program website.
